# Comprehensive intervention for reducing suicidal ideation and depressive symptoms in adolescents with non-suicidal self-injury: a study of Internet-delivered and offline dialectical behavior therapy

**DOI:** 10.3389/fpsyt.2026.1777266

**Published:** 2026-03-13

**Authors:** Yanping Zhang, Jixuan Hou, Meiqi Guo, Yue Zhou, Xue Zhang, Chuansheng Wang, Fang Yan

**Affiliations:** 1 Department of Nursing, Henan Mental Hospital, The Second Affiliated Hospital of Xinxiang Medical University, Xinxiang, Henan, China; 2 Henan Collaborative Innovation Center of Prevention and Treatment of Mental Disorder, Xinxiang, Henan, China; 3 Department of Nursing, Xinxiang Medical University, Xinxiang, Henan, China; 4 Department of Psychology, Xinxiang Medical University, Xinxiang, Henan, China

**Keywords:** adolescents, comprehensive intervention, dialectical behavior therapy, Internet-delivered, non-suicidal self-injury, offline-DBT, rehabilitation

## Abstract

**Objective:**

This study aims to evaluate the efficacy of a comprehensive intervention—Internet-integrated and offline dialectical behavior therapy (DBT)—in reducing suicidal ideation and depressive symptoms and improving cognitive emotion regulation among adolescent non-suicidal self-injury (NSSI).

**Methods:**

Adolescent patients with NSSI (*n* = 120) who were discharged from the follow-up system of The Second Affiliated Hospital of Xinxiang Medical University were enrolled in this study (from September 2022 to October 2024).The control group received Internet-delivered DBT training, while the experimental group received Internet-delivered and offline-DBT sessions. The intervention lasted for 1 year. All participants completed the following questionnaires: Self-Rating Idea of Suicide Scale (SIOSS), Cognitive Emotion Regulation Questionnaire (CERQ-C), and Montgomery–Asberg Depression Rating Scale (MADRS) to evaluate suicidal ideation, cognitive–emotional regulation, and depressive symptoms. Assessments were conducted at baseline, 6 months, and 12 months. Repeated-measures ANOVA was employed to compare the health status of the two groups at different time points.

**Results:**

In the control group (*n* = 55) and the experimental group (*n* = 53), the main effects of the SIOSS optimistic and concealing factor, total score, CERQ-C dimension scores, and MADRS scores were found to be significant (*P* < 0.05). Additionally, the main effects of time on SIOSS factors and total score, CERQ-C dimension scores, and MADRS scores were significant (*P* < 0.05). Furthermore, there was an interactive effect between groups and time on the SIOSS sleep and concealing factor scores, CERQ-C self-blame, contemplation, active refocus, refocus plans, and active reappraisal scores (*P* < 0.05).

**Conclusions:**

Comprehensive intervention effectively mitigates suicidal ideation, alleviates depressive symptoms, enhances cognitive emotion regulation ability, and improves the quality of life in adolescents with NSSI.

## 1 Introduction

Non-suicidal self-injury (NSSI) refers to a series of repetitive, intentional, and direct self-inflicted harm behaviors performed by individuals without suicidal intent, which are socially unacceptable and constitute a critical predictor of suicide mortality (1). Studies have reported that the prevalence of NSSI behaviors among adolescent patients with depressive disorders in China ranges high, from 44% to 61.2%. The global prevalence rate is 19.5%, which is an increasing trend (2). Currently, clinical practice still lacks specific and efficacious intervention strategies for alleviating suicidal ideation and depressive symptoms in this population (3). Spanish researchers argue that Internet-integrated intervention exerts a favorable impact on NSSI treatment outcomes (4). Germany and 11 other countries have issued official intervention guidelines for NSSI, with dialectical behavior therapy (DBT) designated as the primary intervention of choice in psychotherapeutic interventions (5). DBT emphasizes modifying behaviors and regulating emotions through the dialectical balance and integration of acceptance and change, with its core curriculum comprising four key skill modules: mindfulness skills, distress tolerance skills, emotion regulation skills, and interpersonal effectiveness skills—all of which are targeted at improving emotional regulation ability and alleviating depressive and suicidal symptoms (6). American scholar Kothgassner et al. (7) argued that DBT can reduce patients' suicidal ideation and self-injury behavior. However, treatment outcomes remain suboptimal due to such barriers as prolonged treatment duration, patients’ internalized stigma, and limited access to clinical services, whereas the integrated intervention model combining Internet-integrated and offline intervention can effectively address these issues. Internet-integrated intervention is gaining wider use in chronic disease management, and studies have validated its effectiveness in such specialized areas as stoma care and diabetes management. (8, 9). With the support of the “HeNan Province Psychological Assistance Cloud Platform”, an integrated Internet-delivered and offline-DBT comprehensive intervention is adopted to reduce patients' suicidal ideation, improve their emotional regulation ability, alleviate depressive symptoms, provide patients with accessible and efficient services, and facilitate the development of continuous care services in psychiatric hospitals.

## 2 Materials and methods

### 2.1 Participants

**Figure 1:**
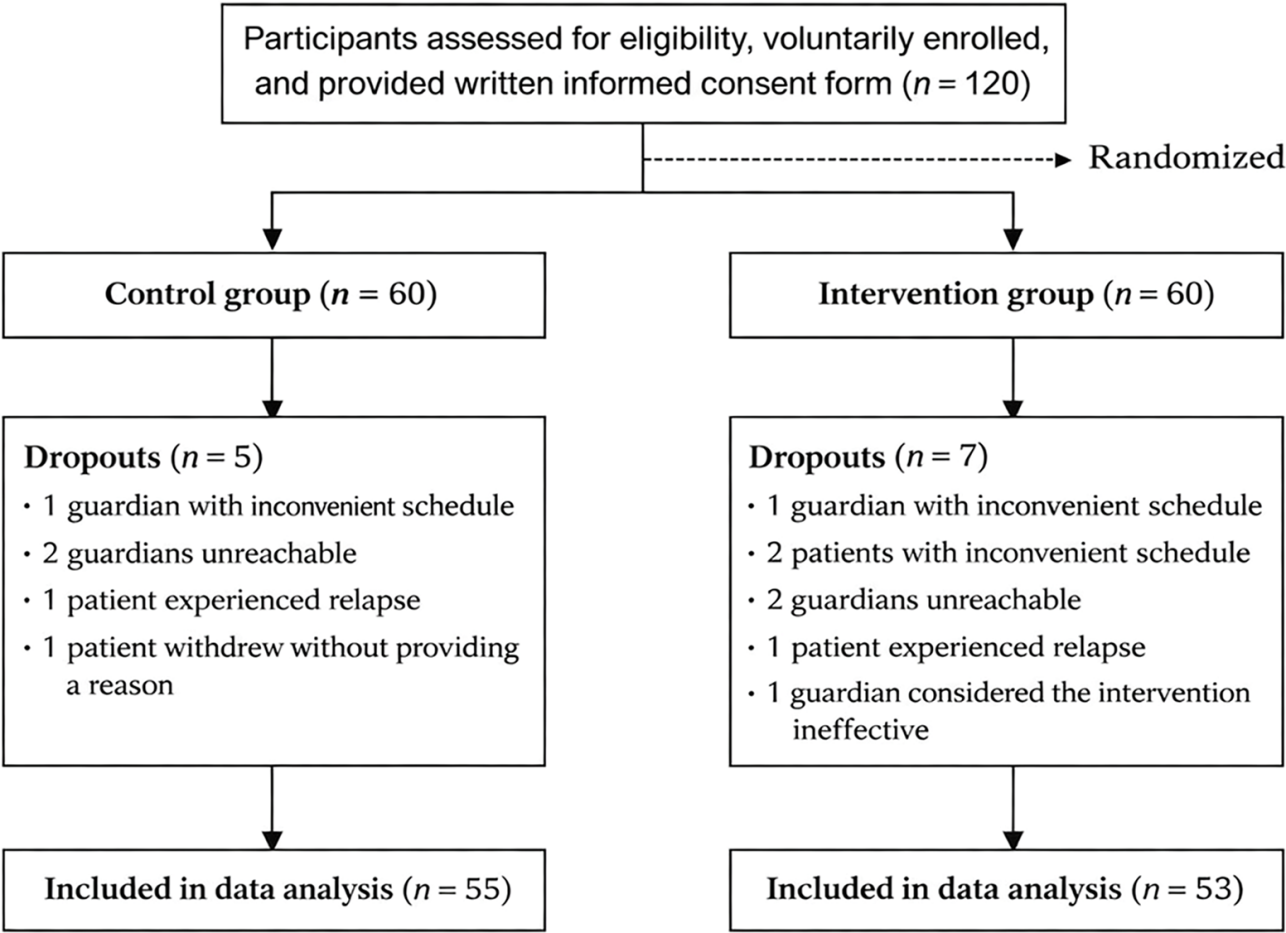
Flow diagram of participant randomization.

Adolescent patients with NSSI (*n* = 120) who were discharged from the follow-up system of The Second Affiliated Hospital of Xinxiang Medical University were enrolled in this study (from September 2022 to October 2024). A statistician uninvolved in the participants’ recruitment or assessment generated a randomization sequence using a computer-generated random number table, and the participants were randomly assigned to a control group (60 individuals) or an experimental group (60 individuals) (see **Figure 1**). The purpose and the significance of the project were explained to the research subjects and their guardians. Written informed consent was obtained, and skills training agreement and crisis plan were signed.

Inclusion criteria: (i) To diagnose depression according to internationally recognized diagnostic criteria (the Diagnostic and Statistical Manual of Mental Disorders, Fifth Edition (DSM-V), (ii) age ranging from 12 to 18 years, (iii) at least one episode of NSSI occurring during hospitalization, (iv) educational level of junior high school or above, with intact cognitive comprehension, adequate verbal communication skills, and the ability to fully articulate personal emotions, (v) demonstrated proficiency in operating the Psychological Assistance Cloud Platform System, and (vi) provision of written informed consent to be randomized, to participate in the study, and to complete pre-intervention and post-intervention assessments.

Exclusion criteria: (i) The Montgomery–Asberg Depression Rating Scale (MADRS) scores are ≥35, indicating that the patients are assessed as having severe depression (10), (ii) patients have major psychological or neurological comorbidities, (iii) patients have an education level below primary school or are unable to use Internet-based systems, and (iv) individuals have a background in psychology. This study is based on a study approved by the Ethics Committee of Second Affiliated Hospital of XinXiang Medical University on August 16, 2022. Its ethical code is XYEFYLL-2022-48.

### 2.2 Randomization and allocation concealment

An independent statistician generated the randomization sequence using a computer-generated random number table. Allocation concealment was strictly implemented by placing the randomization list in sequentially numbered, opaque, sealed envelopes, which were kept by an independent research coordinator who took no part in the clinical procedures. These envelopes were only opened after the participants completed the baseline assessment to ensure the integrity of allocation. A single-blind design was adopted in this study: the outcome assessors were blinded to the participants’ group assignment, while participants and intervention providers could not be blinded due to the inherent nature of the intervention.

### 2.3 Measures

#### 2.3.1 Establishment of training team

The DBT training team was composed of professional technical personnel from the hospital, including eight rehabilitation skills trainers, four psychiatrists, two case managers, two psychological counselors, and eight psychiatric nurses. Rehabilitation skills trainers were required to complete standardized skills training and obtain hospital-accredited rehabilitation training certifications. The participants were divided into eight groups (six to eight cases). The rehabilitation skills trainers were split into four groups, each composed of two members (lead trainer and assistant trainer). Each DBT training team comprised two rehabilitation skills trainers, one psychiatrist, and two psychiatric nurses; additionally, one psychological counselor was assigned to oversee two groups.

Training sessions were conducted twice weekly, with each session lasting 2 h. Psychiatrists were responsible for participant recruitment, diagnostic assessment, pharmacotherapy administration, and outcome scale evaluation. Psychological counselors delivered online psychological counseling services, while psychiatric nurses undertook telephone follow-ups and risk monitoring. Rehabilitation skills trainers communicated the training protocols to adolescent patients with NSSI, with the aim of establishing a collaborative commitment and a therapeutic training alliance.

#### 2.3.2 Establishment of four-skill intervention plans for DBT training

Adolescent patients with NSSI completed the full suite of skill acquisition within 6 months (24 weeks). Within the 1-year treatment protocol, therapists delivered the training content on a repeated basis. The DBT training included four core modules: mindfulness skills (2 weeks), distress tolerance skills (6 weeks), emotion regulation skills (7 weeks), and interpersonal effectiveness skills (5 weeks) (see **Supplementary Materials**).

Each training session had four components: (a) opening session, (b) review and discussion of homework assignments from the previous session, (c) didactic teaching of new content, and (d) closing session.

### 2.4 Intervention methods for the control group

The participants accessed a remote intervention platform, which the rehabilitation skills trainers managed via a video-based format. Reminder (10-min) was issued prior to each session, after which the participants joined by clicking the designated link. The trainers configured platform permissions according to the course schedule and delivered training with group teaching. Synchronous online video demonstrations enabled the trainers to assist the lead instructor in guiding the participants to complete the training tasks in real time.

### 2.5 Intervention methods for the experimental group

**Figure 2:**
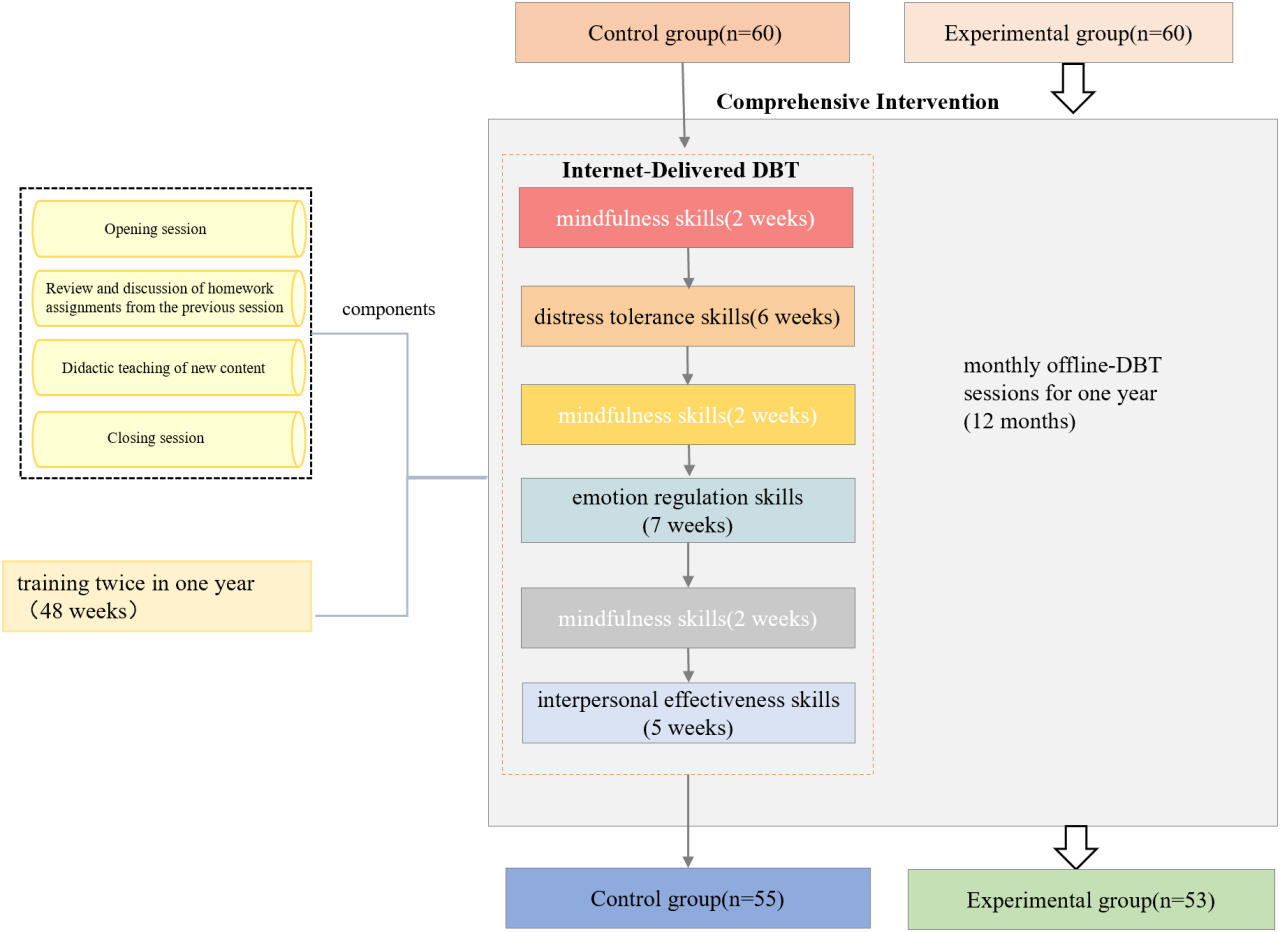
Intervention methods.

The experimental group received the control group intervention, supplemented with monthly offline-DBT sessions for 1 year (12 months). The offline-DBT session contains four core modules on mindfulness skills, distress tolerance skills, emotion regulation skills, and interpersonal effectiveness skills (which lasted for 2 h). The face-to-face sessions include guiding, observation, and communication between rehabilitation trainers and patients (see **Figure 2**).

### 2.6 Research methods

#### 2.6.1 General information

Patient sociodemographic survey included gender, age, education level, illness duration, and number of hospitalizations.

#### 2.6.2 Self-Rating Idea of Suicide Scale

The Self-Rating Idea of Suicide Scale (SIOSS) (11, 12) was used to evaluate the participants' suicidal ideation. This 26-item scale comprises four dimensions (despair, optimistic, sleep, and concealing), with total scores representing the sum of all dimension scores—higher scores indicate a more severe suicidal ideation. The SIOSS demonstrated good reliability, with Cronbach’s *α* coefficient of 0.906, split-half reliability of 0.814, and retest reliability of 0.860.

#### 2.6.3 Cognitive emotion regulation questionnaire

The emotional cognitive level and strategies of patients after experiencing adverse life events were assessed using 36 items, including nine scales (13): self-blame, contemplation, catastrophization, blaming others, acceptance, positive refocusing, replan, positive reappraisal, and rational analysis. The Likert five-point scoring ranges from 1, representing “never”, to 5, representing “always”. Cronbach’s *α* coefficient for the total scale was 0.810, and those for the subscales ranged from 0.480 to 0.910. The retest reliability for the total scale was 0.560, and those for the subscales ranged from 0.360 to 0.690. The average inter-item correlation for the total scale was 0.100, and those for the subscales ranged from 0.190 to 0.710. Higher scores on each subscale indicated a stronger tendency toward the corresponding emotion regulation strategy.

#### 2.6.4 Montgomery and Asberg depression rating scale

The Chinese version was developed by Zhong Baoliang et al. (14, 15), with 10 items. All items were scored using a seven-point Likert rating system (0 represents “normal” and 6 represents “severe depression”). Higher scores indicate more severe depressive symptoms. For efficacy evaluation, a total score reduction ≥50% was considered effective compared to baseline; total score ≤10 indicates clinical recovery or remission. Cronbach’s *α* was 0.825, the retest reliability for the total scale was 0.737. It assessed adolescent depression intervention outcomes and sensitively reflected changes in depressive symptoms.

### 2.7 Data collection methods

Members of the research introduced the research purpose, duration, and procedures to patients and guardians after enrollment. General information, SIOSS, Cognitive Emotion Regulation Questionnaire (CERQ-C), and Montgomery and Asberg Depression Rating Scale (MADRS) were collected through the platform before intervention and after 6 and 12 months of intervention. The questionnaires required to be completed within 48 h; the platform settings prohibited submissions with missing items. Two reminders were sent, with uncompleted forms deemed invalid.

### 2.8 Quality control

1. DBT rehabilitation training was delivered through a standard protocol; the staff completed mandatory unified training, with only passers eligible for intervention involvement.
2. Scales and questionnaires were distributed via the platform; upon completion, their completeness and key information were verified promptly, followed by a full review of data consistency.
3. The training participants earned rewards via a points system.
4. Offline session transportation costs were reimbursed monthly.

### 2.9 Statistical analysis

Data were analyzed using the SPSS 26.0. Normally distributed continuous data were summarized as mean ± standard deviation (mean ± SD), with inter-group comparisons performed using independent-samples *t*-tests. Repeated-measures analysis of variance was applied to analyze the longitudinal scale scores of the two groups at multiple time points—baseline and post-intervention. Missing data were handled using mean imputation. Repeated measures analysis of variance (ANOVA) was used to analyze scale scores at multiple time points before and after intervention. For significant main or interaction effects, *post-hoc* pairwise comparisons with Bonferroni correction were conducted to examine within-group differences across time points (baseline, 6 months, and 12 months). The significance level was adjusted to *P* < 0.016 (0.05/3) to account for multiple comparisons.

This study employed the per-protocol set (PPS) for primary analysis, excluding participants who were lost to follow-up, withdrew from the study, or failed to complete at least one post-intervention assessment. No intention-to-treat (ITT) analysis was performed, as mean imputation was used to address data missing data and was only applicable to participants who completed the primary assessment time points.

## 3 Results

### 3.1 Participants’ characteristics

A total of 55 patients were in the control group (five dropped out) and 53 in the experimental group (seven dropped out), with a scale recovery rate of 91.67% and 88.33%, respectively. No statistically significant differences (*P* > 0.05) were observed between the two groups in gender, age, education level, disease duration, and hospitalization frequency (see **Table 1**).

**Table 1:** Comparison of general information between the two groups.

Variables	Control group (n=55)	Experimental group (n=53)	χ^2^(*t)* value	*P* value
Age (years)	14.98±1.42	15.20±1.64	0.735^a^	0.464
Sex (female/male)	32/23	33/20	0.188	0.665
Grade [n(%)]			0.131	0.896
Junior	25 (45.4)	24 (45.2)		
Senior	27 (49.1)	25 (47.1)		
Above collage	3 (5.4)	4 (7.5)		
Duration of illness (years)	1.76±0.82	1.79±0.84	0.167^a^	0.867
Number of hospitalizations [n(%)]			0.514	0.608
Once	37 (67.2)	33 (62.3)		
Twice	15 (27.3)	17 (32.1)		
More	3 (5.4)	3 (5.6)		

a t (t-test).

### 3.2 Comparison of SIOSS scores between two groups

The main effects of group on SIOSS optimistic, concealing factors, and total score were significant (*P* < 0.05); the main effects of time on all SIOSS factors and total score were also significant (*P* < 0.05).

**Figure 3:**
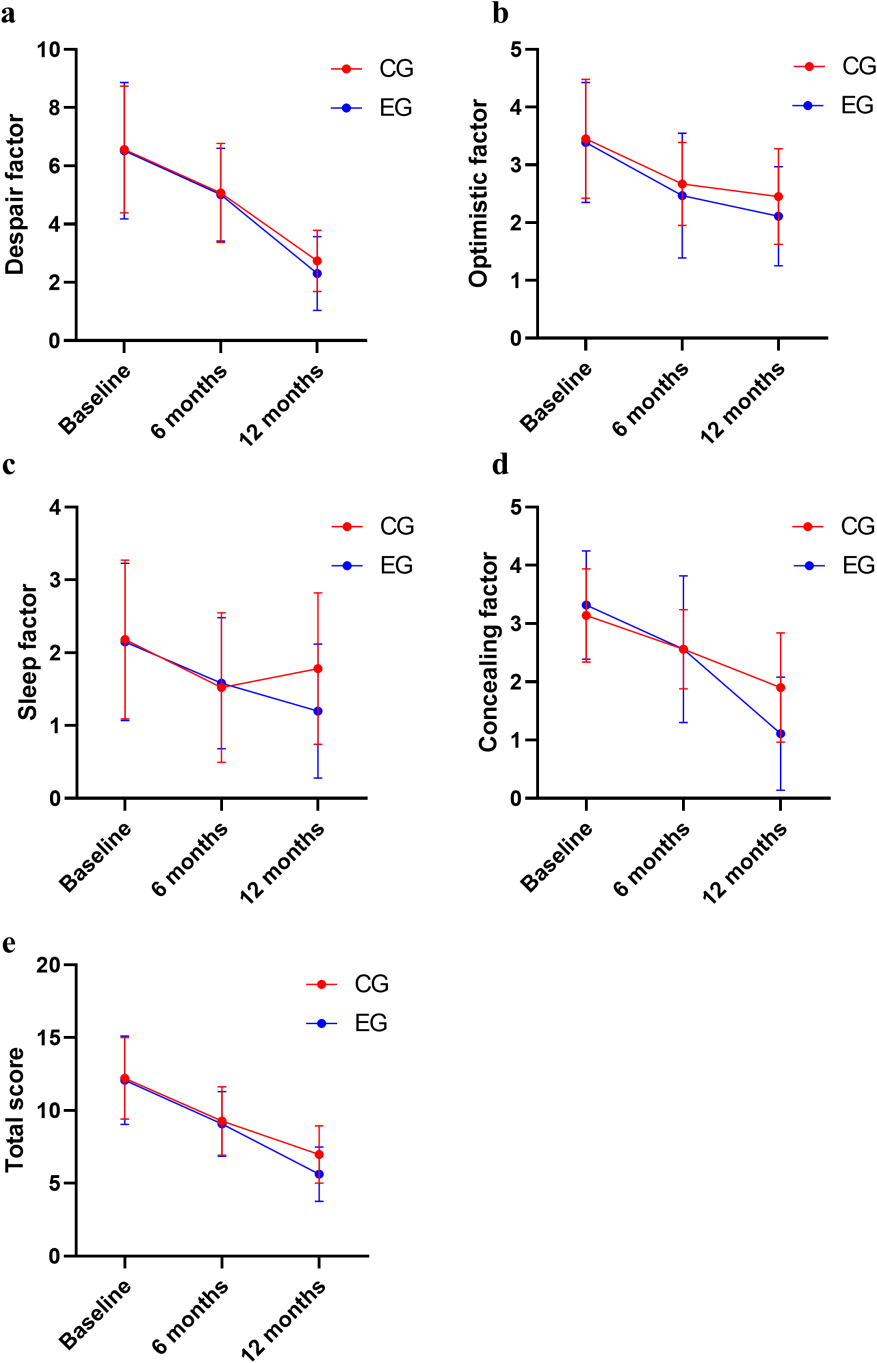
Comparison of SIOSS scores between two groups. **(a)** Comparison of despair score; **(b)** Comparison of optimistic score; **(c)** Comparison of sleep score; **(d)** Comparison of concealing score; **(e)** Comparison of SIOSS total score.

There was an interactive effect between groups and time on the SIOSS sleep and concealing factor scores (*P* < 0.05). No statistically significant differences were found in SIOSS factors or total score between the two groups at baseline (see **Figure 3**, **Table 2**).

**Table 2:** Comparison of SIOSS scores between the two groups (Mean ± SD).

Variables	Group	Before intervention	After intervention		*F*_time_	*F _group_*	*F _interactive_*
			6 months	12 months			
Despair factor	Experimental group	6.52±2.34	5.01±1.59	2.30±1.27	158.377 *P* < 0.001	0.709 *P* = 0.402	0.509 *P* = 0.602
	Control group	6.56±2.18	5.07±1.70	2.74±1.05			
					*η²_p_* = 0.599(95% CI 0.524-0.671)	*η²_p_* = 0.007(95% CI 0.000-0.071)	*η²_p_* = 0.005(95% CI 0.000-0.050)
Optimistic factor	Experimental group	3.39±1.04	2.47±1.08	2.11±0.86	43.718 *P* < 0.001	3.580 *P* = 0.061	0.621 *P* = 0.538
	Control group	3.45±1.03	2.67±0.72	2.45±0.83			
					*η²_p_* = 0.292(95% CI 0.199-0.390)	*η²_p_* = 0.033(95% CI 0.000-0.127)	*η²_p_* = 0.006(95% CI 0.000-0.053)
Sleep factor	Experimental group	2.15±1.08	1.58±0.90	1.20±0.92	14.604 *P* < 0.001	2.518 *P* = 0.116	3.099 *P* = 0.047
	Control group	2.18±1.09	1.52±1.03	1.78±1.04			
					*η²_p_* =0.121(95% CI 0.055-0.212)	*η²_p_* = 0.023(95% CI 0.000-0.109)	*η²_p_* = 0.028(95% CI 0.004-0.093)
Concealing factor	Experimental group	3.32±0.93	2.56±1.26	1.11±0.97	97.383 *P* < 0.001	3.323 *P* = 0.071	8.670 *P* < 0.001
	Control group	3.14±0.80	2.56±0.68	1.90±0.94			
					*η²_p_* = 0.479(95% CI 0.392-0.561)	*η²_p_* = 0.030(95% CI 0.000-0.123)	*η²_p_* = 0.076(95% CI 0.026-0.159)
Total score	Experimental group	12.07±3.03	9.07±2.22	5.62±1.87	163.815 *P* < 0.001	4.016 *P* = 0.048	2.309 *P* = 0.102
	Control group	12.20±2.81	9.27±2.36	6.98±1.97			
					*η²_p_* = 0.607(95% CI 0.533-0.676)	*η²_p_* = 0.037(95% CI 0.001-0.134)	*η²_p_* = 0.021(95% CI 0.002-0.084)

### 3.3 Comparison of CERQ-C scores between two groups

**Figure 4:**
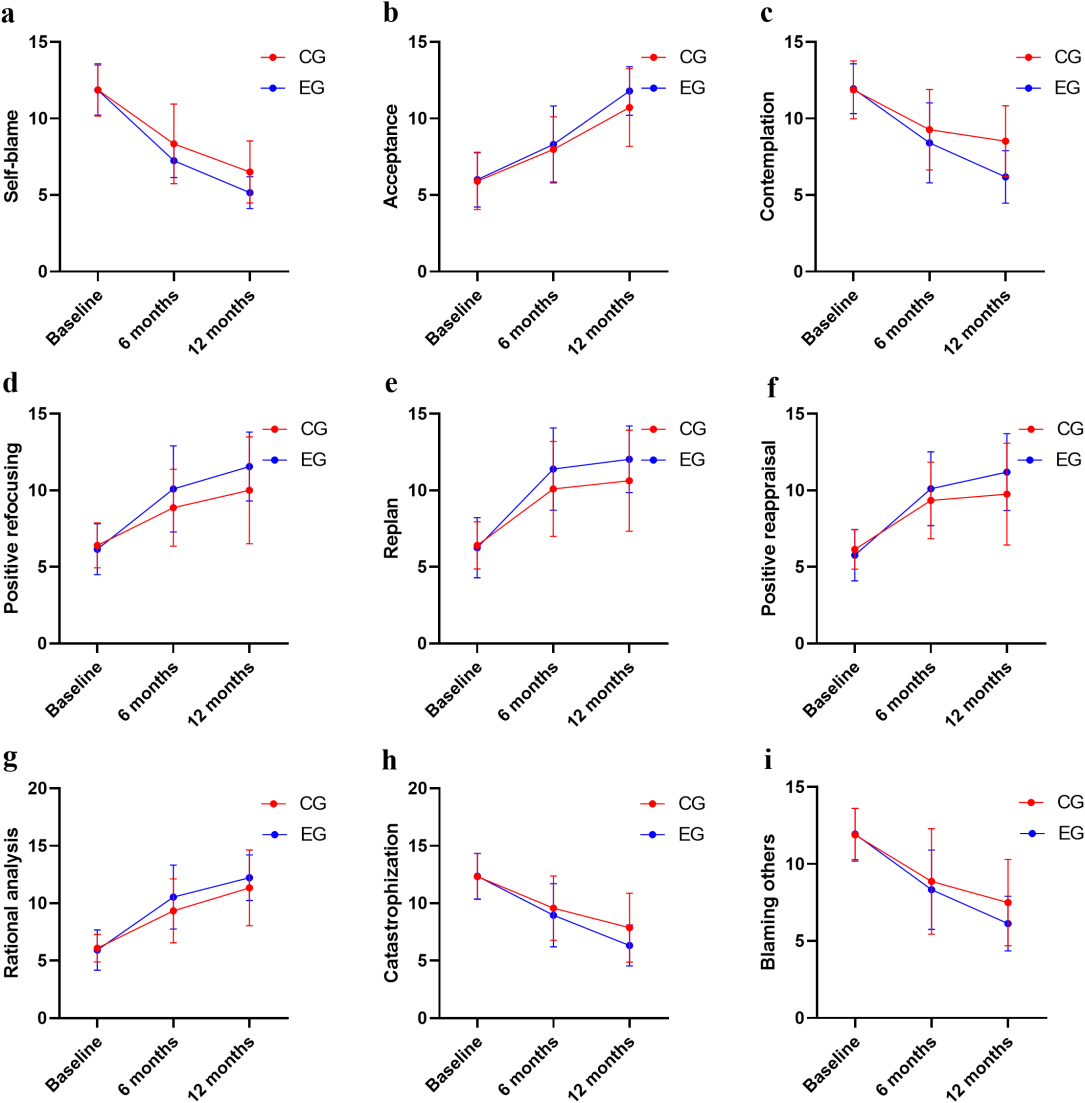
Comparison of CERQ-C scores between two groups. **(a)** Comparison of self-blame score; **(b)** Comparison of acceptance score; **(c)** Comparison of contemplation score; **(d)** Comparison of positive refocusing score; **(e)** Comparison of replan score; **(f)** Comparison of positive reappraisal score; **(g)** Comparison of rational analysis score; **(h)** Comparison of catastrophization score; **(i)** Comparison of blaming others score.

The main effects of group and time on all CERQ-C dimensions were significant (*P* < 0.05). There was an interactive effect (*P*  < 0.05) between groups and time on CERQ-C self-blame, contemplation, positive refocusing, replan, and positive reappraisal scores. No statistically significant differences were observed in CERQ-C dimension scores between the two groups at baseline (see **Figure 4**, **Table 3**).

**Table 3:** Comparison of CERQ-C scores between the two groups (Mean ± SD).

Variables	Group	Before intervention	After intervention		*F*_time_	*F _group_*	*F _interactive_*
			6 months	12 months			
Self-blame	Experimental group	11.86±1.64	7.24±1.10	5.15±1.04	357.001	14.450	4.855
	Control group	11.87±1.71	8.34±2.60	6.50±2.03	*P* < 0.001	*P* < 0.001	*P* = 0.009
					*η²_p_* = 0.771(95% CI 0.726-0.812)	*η²_p_* = 0.120(95% CI 0.031-0.246)	*η²_p_* = 0.044(95% CI 0.009-0.116)
Acceptance	Experimental group	6.00±1.79	8.30±2.52	11.79±1.59	176.124	4.219	1.607
	Control group	5.90±1.86	7.98±2.12	10.72±2.54	*P* < 0.001	*P* = 0.042	*P* = 0.203
					*η²_p_* = 0.624(95% CI 0.552-0.691)	*η²_p_* = 0.038(95% CI 0.001-0.137)	*η²_p_* = 0.015(95% CI 0.001-0.073)
Contemplation	Experimental group	11.94±1.63	8.41±2.62	6.18±1.72	134.595	15.656	9.229
	Control group	11.87±1.89	9.27±2.63	8.52±2.31	*P* < 0.001	*P* < 0.001	*P* < 0.001
					*η²_p_* = 0.559(95% CI 0.478-0.639)	*η²_p_* = 0.129(95% CI 0.037-0.257)	*η²_p_* = 0.080(95% CI 0.026-0.169)
Positive refocusing	Experimental group	6.16±1.67	10.09±2.81	11.56±2.25	98.212	9.021	4.164
	Control group	6.40±1.47	8.87±2.51	10.00±3.49	*P* < 0.001	*P* = 0.003	*P* = 0.017
					*η²_p_* = 0.481(95% CI 0.392-0.571)	*η²_p_* = 0.078(95% CI 0.010-0.194)	*η²_p_* = 0.038(95% CI 0.005-0.111)
Replan	Experimental group	6.26±1.97	11.39±2.69	12.03±2.18	158.456	5.488	3.285
	Control group	6.40±1.54	10.10±3.11	10.63±3.30	*P* < 0.001	*P* = 0.021	*P* = 0.039
					*η²_p_* = 0.599(95% CI 0.527-0.668)	*η²_p_* = 0.049(95% CI 0.001-0.156)	*η²_p_* = 0.030(95% CI 0.004-0.096)
Positive reappraisal	Experimental group	5.77±1.67	10.11±2.41	11.20±2.52	116.163	4.949	4.161
	Control group	6.14±1.29	9.34±2.51	9.76±3.33	*P* < 0.001	*P* = 0.028	*P* = 0.017
					*η²_p_* = 0.523(95% CI 0.438-0.605)	*η²_p_* = 0.045(95% CI 0.001-0.146)	*η²_p_* = 0.038(95% CI 0.005-0.110)
Rational analysis	Experimental group	5.92±1.75	10.54±2.79	12.22±1.98	155.980	6.605	2.291
	Control group	6.09±1.20	9.34±2.79	11.34±3.30	*P* < 0.001	*P* = 0.016	*P* = 0.104
					*η²_p_* = 0.595(95% CI 0.516-0.671)	*η²_p_* = 0.054(95% CI 0.003-0.163)	*η²_p_* = 0.021(95% CI 0.001-0.086)
Catastrophization	Experimental group	12.35±1.99	8.96±2.76	6.32±1.79	127.406	6.607	2.903
	Control group	12.32±2.01	9.58±2.81	7.87±3.00	*P* < 0.001	*P* = 0.012	*P* = 0.057
					*η²_p_* = 0.546(95% CI 0.467-0.624)	*η²_p_* = 0.059(95% CI 0.004-0.169)	*η²_p_* = 0.027(95% CI 0.003-0.093)
Blaming others	Experimental group	11.94±1.66	8.33±2.58	6.13±1.77	138.053	4.247	2.587
	Control group	11.89±1.71	8.87±3.42	7.49±2.80	*P* < 0.001	*P* = 0.042	*P* = 0.078
					*η²_p_* = 0.566(95% CI 0.485-0.645)	*η²_p_* = 0.039(95% CI 0.001-0.138)	*η²_p_* = 0.024(95% CI 0.002-0.090)

### 3.4 Comparison of MADRS scores between two groups

**Figure 5:**
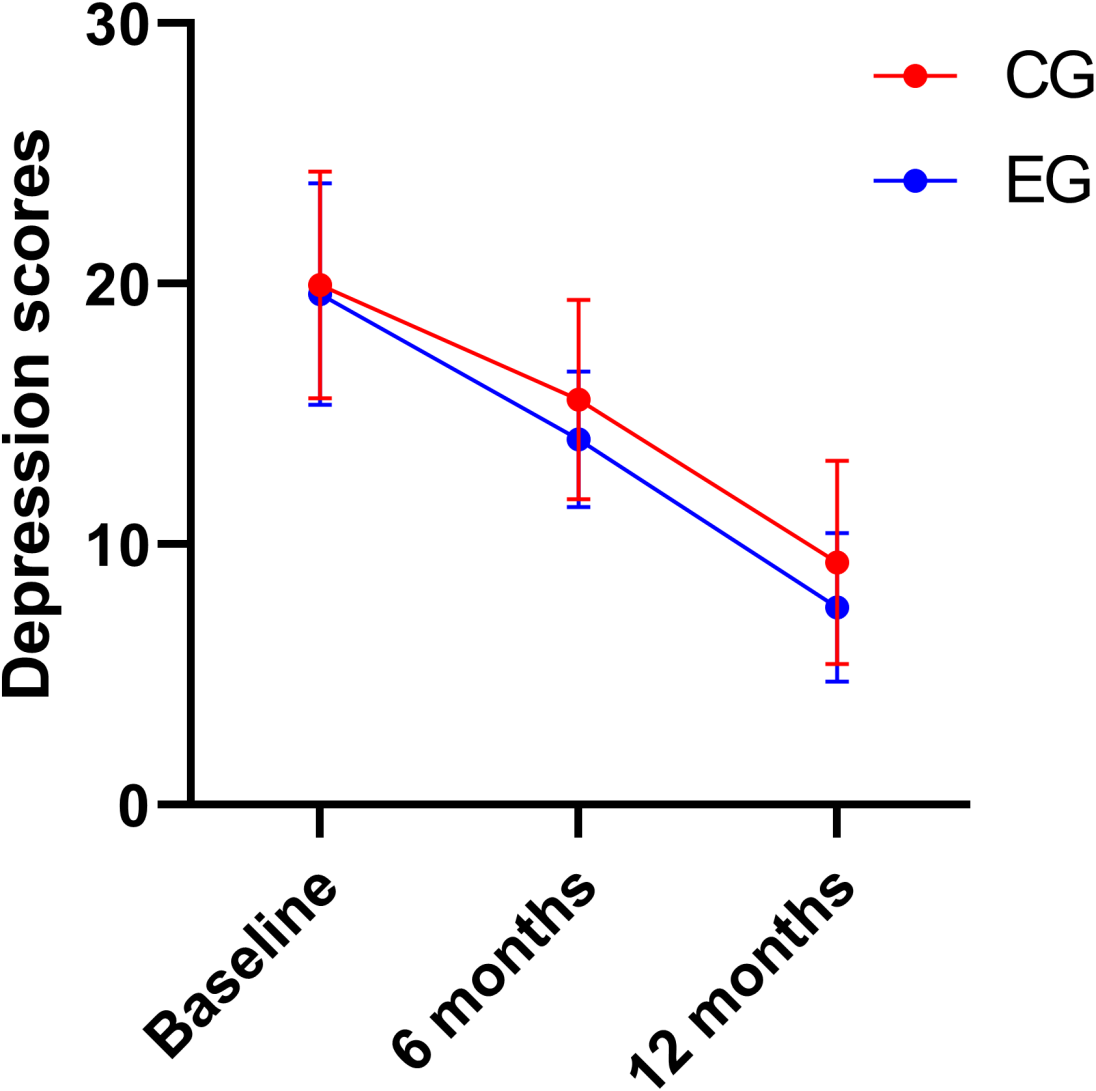
Comparison of MADRS scores between two groups.

The main effects of group and time on MADRS scores were significant (*P* < 0.05). There was no interaction between groups and time on MADRS scores (*P* > 0.05).At baseline, no significant differences were noted in MADRS scores between the two groups (see **Figure 5**, **Table 4**).

**Table 4:** Comparison of MADRS scores between the two groups (Mean ± SD).

Variables	Group	Before intervention	After intervention		*F*_time_	*F _group_*	*F _interactive_*
			6 months	12 months			
Depression scores	Experimental group	19.62±4.27	14.03±2.60	7.60±2.86	292.850	6.603	1.270
	Control group	19.96±4.35	15.56±3.83	9.32±3.92	*P* < 0.001	*P* = 0.012	*P* = 0.283
					*η²_p_*=0.734(95%CI 0.680-0.784)	*η²_p_* = 0.059(95% CI 0.004-0.169)	*η²_p_*=0.012(95%CI 0.001-0.067)

## 4 Discussion

### 4.1 The comprehensive intervention shows a more obvious improvement trend in reducing suicidal ideation

Suicidal ideation in NSSI adolescents arises from chronic emotional suppression and insufficient coping skills, with depression including crying spells, self-rejection, feelings of worthlessness, and social isolation (16). Perceptions of life as cruel, combined with inadequate understanding and responses from parents and relatives, further induce cognitive dissonance and suicidal ideation (17). This study indicated that the experimental group exhibited lower SIOSS scores than the control group following 12 months of intervention, suggesting that integrated online–offline training may show a more obvious improvement trend in reducing patients’ suicidal ideation—which is consistent with the findings of Kaess et al. (18) and Mehlum et al. (19). Through comprehensive intervention, patients actively engaged in and cooperated with training sessions; additionally, they communicated directly with clinicians monthly, with key components including simulated scenario re-demonstrations, real-time interaction, and progress monitoring. Compared with those only receiving online training, patients in the comprehensive intervention group received timely clarification, correction, and guidance for their questions, which may have improved their comprehension and operational accuracy and potentially enhanced treatment trust and adherence. These outcomes may be related to the additional support brought by face-to-face communication. Active mindfulness skill training enabled patients to consciously observe and experience events with curiosity and non-judgmental awareness, focusing on describing current situations accurately without bias or distortion. They developed the belief that all challenges have underlying causes, sought targeted solutions, alleviated emotional distress, and rediscovered their authentic selves.

Moreover, distress tolerance skills teach patients that pain is an inherent part of life that cannot be avoided or eliminated. By learning to tolerate pain temporarily, patients may become better equipped to initiate positive changes. As emphasized in sessions, when confronted with adversity, patients should step back and pause to regain emotional control through deep breathing before attempting to objectively assess the situation without rushing to conclusions—thereby aiming to address problems accurately and effectively. When patients experience overwhelming emotions and are tempted to engage in self-harm for relief, strategies with cold water facial washing, ice holding, distraction, and self-soothing may help rapidly alleviate suicidal ideation. In interpersonal effectiveness training, instructors seek to enhance patients’ communication skills via simulated social scenarios—for instance, practicing assertive refusal in simulated scenario enables patients to prioritize their needs through clear self-expression while aiming to preserve their self-esteem and interpersonal relationships. Positive interpersonal connections may help redirect patients’ potential negative behaviors toward constructive alternatives, which could contribute to reducing suicidal ideation. Compared with the intervention received by the control group, comprehensive intervention, which combines online flexibility with offline pertinence, appears to address the shortcomings of single online interventions (inadequate real-time interaction, weak emotional connection), potentially suggesting that it may show a more obvious improvement trend in enhancing DBT’s effect on reducing suicidal ideation. This observation also aligns with the findings of Santamarina-Perez et al. (20) and Berk et al. (21), which primarily aimed to reduce suicidal ideation and behavior.

### 4.2 Comprehensive intervention improves the emotional regulation cognitive strategies

The main effects of group and time on all CERQ-C dimensions were significant in the experimental group (*P* < 0.05) after 6 and 12 months. This finding may be related to the additional support brought by face-to-face communication between rehabilitation skills trainers and patients, which provides patients with more personalized and deep support and further improves their intervention adherence—for instance, emotions such as anger and sadness often arise from reactions to one’s own thoughts and interpretations of events rather than the objective facts. By guiding patients to verify the facts firstly and teaching them how to develop active problem-solving strategies tailored to the actual situation, maladaptive emotions may be alleviated.

Patients are guided to ask themselves three questions when confronted with anger-triggering events: Is the perception of the event consistent with the facts? Is the anger an effective response? Would it be better to refrain from impulsive actions and first deliberate on coping approaches? They are encouraged to try to change their thought and practice antagonistic coping behaviors to anger through repeated training, thereby helping to regulate and stabilize their emotions, such as perspective thinking or deep breathing. As positive emotions increased, patients reduced overfocus on worries, created a pleasure checklist, and completed one to two daily items (e.g., dancing, listening to music, flower arranging). Defining core values and setting small, specific life goals allowed the patients to actively refocus, plan, and evaluate emotional responses as well as analyze problems rationally. Asarnow et al. (22) and Yeo et al. (23) argued that DBT training can alleviate negative emotions and expand positive emotion-regulation strategies in adolescents with NSSI.

### 4.3 Comprehensive intervention alleviates depressive symptom

Various studies have revealed that DBT training can significantly reduce depression symptoms in adolescents with NSSI (24, 25). Research has indicated that adolescent depression patients have difficulty regulating their emotions and tend to adopt negative, avoidance, and silent attitudes when facing the disease. Depressive emotions are significantly positively correlated with adolescent NSSI (26). This study reveals that the MADRS score of the experimental group was lower than that of the control group 6 and 12 months after comprehensive intervention (*P*  < 0.05). This finding may be related to the additional support brought by face-to-face communication between rehabilitation trainers and patients, which rendered the training program more targeted and effective. The training speed was adjusted immediately through real-time interaction and monitoring to ensure the best effect. This interaction and monitoring may not be timely and comprehensive in online intervention. However, face-to-face communication and physical contact may effectively help patients express their inner feelings, release negative emotions, and alleviate depressive symptoms.

Emotional regulation training may help patients build and accumulate positive emotions and practice self-regulation, for example, by identifying, labeling, and pinpointing the triggers of depressive feelings. Patients first learn that low mood cannot be effectively alleviated in isolation; they then set emotion-regulation goals and take concrete steps to accept their feelings and reduce depressive symptoms (27). Through mindfulness training, it is determined that the life goal is to reduce pain and increase happiness, telling patients to live in the present and be mindful of their thoughts, which may exert a positive impact on their depressive emotions. Individual negative interpersonal relationships are an important cause of anxiety and depression in patients (28). In this study, interpersonal efficacy skills training was conducted to inform patients to adopt a middle-ground approach in interpersonal communication, accept reality, and strive to change their current situation. For patients reluctant to communicate, nonverbal responses (e.g., facial expressions, written notes) and efforts to articulate their thoughts are recommended. They should also acknowledge objective facts, verify them without resistance, develop appropriate interpersonal skills, and improve relationships to alleviate depressive symptoms. This study further indicates that DBT training requires experience or skill-building sessions, and the comprehensive intervention may show a more obvious improvement trend in facilitating better intervention outcomes.

### 4.4 Limitations

This study also has several limitations. The experimental group received an additional monthly offline DBT session alongside the basic Internet-delivered intervention identical to that of the control group, leading to unequal total intervention contact time between the two groups. This may affect the accuracy of the analysis, and the influence of time as a confounding variable can be eliminated in the future through a more rigorous experimental design.

## 5 Conclusion

This study demonstrates that the comprehensive intervention shows a more obvious improvement trend in reducing suicidal ideation, enhancing emotional regulation, alleviating depressive symptoms, and improving the quality of life and social functioning in adolescents with NSSI. Due to the small sample size included in this study, it is necessary to expand the research sample for a more comprehensive exploration to further evaluate the effectiveness of online training combined with offline intervention. It is suggested that the national level should increase investment, pay attention to the mental health of adolescents, establish a comprehensive intervention team for NSSI Internet-integrated intervention for adolescents, and set up a full-time Internet intervention team in psychiatric hospitals to comprehensively conduct health management for school educators, parents of patient, non-psychiatric medical staff from parental rearing methods, early intervention, health lectures, school crisis intervention, and move forward as the direction of future research.

## Data Availability

The raw data supporting the conclusions of this article will be made available by the authors, without undue reservation.
