# Supplementary material for: Comprehensive intervention for reducing suicidal ideation and depressive symptoms in adolescents with non-suicidal self-injury: a study of Internet-delivered and offline dialectical behavior therapy
Source: Front Psychiatry. 2026 Mar 13;17:1777266. doi: 10.3389/fpsyt.2026.1777266 (PMC13021827; doi:10.3389/fpsyt.2026.1777266)
Supplement: Supplementary file 1 [file Table1.docx]

**SITable 1.** Mindfulness skills.

| Time (week) | Aim | Intervention content | Intervention forms | Intervener |
| --- | --- | --- | --- | --- |
| One week | Introduction; Goals and Guidelines | Section 1, Objectives of Skills Training:   1. General objectives; 2. Clear goals: Reduce behaviors and increase skills;   (3) Personal goals  Section 2, Skills Training Guidelines:   1. If absent continuously for 4 times, it deemed as suspension; 2. Clarify the requirements for participants to participate in skills training; 3. Clarify the requirements for participants in skills training groups; 4. Students are not allowed to induce others to engage in problematic behavior;   (5) There must be no private relationships between each other outside of training. | Collective teaching | Rehabilitation skills trainer |
| Two weeks | Wisdom mindfulness; Mindfulness "What" skills | Section 1, Wisdom Mindset: Rational mind, emotional mind, wisdom mind. The wisdom mind is the combination of the emotional mind and rational mind, which incorporates intuition into emotional experience and logical analysis.  Section 2: Mastering Your Heart: Observing, describing, and participating. Practice the "What" skill by repeatedly practicing the methods of observation, description, and participation. |  |  |
| Three weeks | Mindfulness "How to Do" Skills | Section 1: Mastering Your Heart: Observing, describing, and participating. Practice the "What" skill by repeatedly practicing the methods of observation, description, and participation.  Section 2: Mastering Your Heart - "How to Do" Skills: Including Not Judging, Focusing on Doing, and Effectively Doing. Moreover, repeatedly practice the "how to do" skill. |  |  |

**SITable 2.** Distress tolerance skills

| Time (week) | Aim | Intervention content | Intervention forms | Intervener |
| --- | --- | --- | --- | --- |
| One week | Crisis survival, advantages and disadvantages analysis | Section 1: The Goal of Enduring Pain:   1. Survive in a crisis without making things worse. 2. Accept the current reality. 3. Obtain freedom.   STOP skills: stop moving, take a step back, objectively observe, act with awareness  Section 2: Advantages and Disadvantages Analysis: Comparing the advantages and disadvantages of acting on emotional impulses and resisting these impulsive behaviors in crises | Collective teaching | Rehabilitation skills trainer |
| Two weeks | Changes body chemistry | Section 1, T: Use cold water to change the temperature of the face;  I: Intense exercise;  P: Respiratory regulation (by slowing down breathing), paired muscle relaxation (muscle relaxation combined with exhalation)  Section 2: Effective Rethinking and Pairing Relaxation, Decomposition, Practice. | Collective/Video  teaching |  |
| Three weeks | Attention diverting, self-soothing, and improving the present | Section 1:   1. Attention diverting: Practice following the 7 steps of shifting attention. 2. Self-soothing: Treat oneself well in a way that comforts, takes care of, peaceful, gentle, and mindful, including using the five senses to soothe oneself and using body scanning meditation for self-soothing   Section 2: Improving the Present: Replace negative events with more positive ones to make the present more positive and tolerable. This is a unique set of strategic activities that break down steps into imagination, meaning, prayer, relaxation, doing one thing once, vacation, and encouragement. | Collective/Video  teaching |  |
| Four weeks | Accept reality | Section 1: Complete Acceptance: It is a complete openness to the truth of reality, guiding members to determine which facts need to be accepted in life. They fill out the complete acceptance exercise form and search for the facts that need to be accepted in their own lives during classes. They practice complete acceptance according to the decomposition steps.  In Section 2, this skill is used to help individuals choose the path of acceptance. Choosing acceptance does not equate to acceptance itself, but rather choosing a direction to move forward. It includes four decomposition steps, namely detecting resistance emotions, calming down and making promises, trying again, and making plans. | Collective teaching |  |
| Five weeks | I am willing; Smiling; A gesture of willingness | Section 1: I am willing: It is a willingness to be fully involved in oneself and participate in the current life. Skills include saying "I am willing" to each situation, replacing my persistence with "I am willing", and taking steps that I am willing to take.  In Section 2: Smiling and a gesture of willingness are the ways in which the body accepts reality. Demonstrate how to relax facial muscles (smiling) and your shoulders, arms, and hands (gesture of willingness). | Collective /Video  teaching |  |
| Six weeks | Mindfulness thoughts | Mindfulness thoughts: The key is to allow your heart to simply let various ideas come and go, without trying to control or change them, and examine them. Mindfulness towards ideas is essential in each step. | Collective teaching |  |

**SITable 3.** Emotion regulation skills.

| Time (week) | Aim | Intervention content | Intervention forms | Intervener |
| --- | --- | --- | --- | --- |
| One week | Understand and name emotions | Understand and name emotions: observe and describe current emotional reactions without judgment, including the function of recognizing emotions and the relationship between difficulty in changing emotions. Learn how to identify emotions and name them in daily life. | Collective teaching | Rehabilitation skills trainer |
| Two weeks | Verify the facts | Section 1: Changing emotional reactions refers to changing unwanted emotions, which includes three parts: verifying facts, opposing behavior, and problem-solving.  Section 2, Fact-checking:  (1) Describe the situation, as well as ideas and interpretations that may trigger emotional reactions;  (2) It is to encourage members to think instead of ideas, interpret, and rewrite facts. |  |  |
| Three weeks | Contrary actions | Opposite behavior = taking actions that are opposite to emotional impulses. Including behavioral activation, exposure therapy, and effective anger therapy, emphasizing learning to identify clues of frustration or anger. |  |  |
| Four weeks | Problem resolving | Problem-solving = avoiding or changing (solving) problem events. Divided into 7 steps:   1. Observe and describe the problem situation; 2. Verify facts to ensure a correct understanding of the problem context; 3. Identify the goal of problem-solving; 4. Brainstorm a large number of solutions; 5. Choose a solution that may work according to the goal; 6. Put the solution into action; 7. Evaluate the results of using this solution. |  |  |
| Five weeks | Accumulate positive emotions (short-term) | Something that pleases oneself and can be done now. Including three steps: creating a positive experience in the present, maintaining awareness of the positive experience, and no longer focusing on one's various worries |  |  |
| Six weeks | Accumulate positive emotions (long-term) | Make some changes to your life, add more positive energy, and form a "life worth living" pattern. Including 7 steps:   1. avoid avoidance; 2. Confirm the values that are important to you; 3. Confirm a currently executable value system; 4. Confirm several goals related to this value system; 5. Choose a currently executable goal; 6. Confirm the action steps to achieve the goal; 7. Now, take an action step. |  |  |
| Seven weeks | Self-control  Responding in advance  PLEASE skills; Emotional mindfulness | 1. Do at least one thing every day; 2. Successfully planned for the purpose; 3. the difficulty gradually increases as time goes by; 4. Proactively facing challenges.   (1) Describe the problem situation;  (2) Deciding which skills to use;  (3) Imagining the situation;  (4) Practice the process of effective coping in the mind;  (5) After the drill, practice to relax  PLEASE  PL: treatment for physical illnesses, E: balanced diet, A: avoidance of mood-altering substances, S: balanced sleep, E: appropriate exercise.  Mindfulness towards current emotions: it means observing, describing, and 'allowing' emotions to come and go truthfully, without judging, suppressing, blocking, or diverting one's attention from emotions. The steps are as follows: observe your own emotions, practice mindfulness using bodily sensations, remember that 'you' are not your emotions, and try to accept your emotions. | Collective /Video  teaching |  |

**SITable 4.** Interpersonal effectiveness skills.

| Time (week) | Aim | Intervention content | Intervention forms | Intervener |
| --- | --- | --- | --- | --- |
| One week | Understand obstacles; Clarify objectives | Section 1: Understand the factors that hinder interpersonal effectiveness and what interpersonal skills you lack. What are the misunderstandings that hinder the effectiveness of the target? What are the misunderstandings about hindering relationships and self-efficacy?  Section 2: Clarify the goals in interpersonal situations and clarify the priority order of interpersonal efficacy in each situation. | Collective teaching | Rehabilitation skills trainer |
| Two weeks | DEAR MAN skills | Goal effectiveness: Effectively maintain your power and expectations as you wish. The decomposition steps are as follows:  D: describes the situation;  E: expresses feelings;  A: clarifies attitude;  R: strengthens the other party;  M: maintains mindfulness;  A: shows confidence;  N: negotiates compromise. |  |  |
| Three weeks | GIVE skills | Relationship efficacy: Maintain relationships and do everything possible to satisfy oneself and others. The decomposition steps are as follows:  G: remains gentle;  I: shows interest;  V: approved others;  E: has a relaxed attitude. |  |  |
| Four weeks | FSAT skills | Self-esteem efficacy: Respect oneself and take action to maintain self-esteem. The decomposition steps:  F: fair treatment;  A: does not apologize excessively;  S: adheres to values;  T maintains sincerity |  |  |
| Five weeks | Evaluation selections | Factors to consider: When making a request or refusal, maintain a firm attitude. The intensity of practicing responding to or rejecting requests from others includes factors such as   1. ability; 2. Priority order; 3. Self-esteem; 4. Power; 5. Permission; 6. Relationship; 7. Long-term and short-term goals; 8. Provide and take; 9. Preparation work in advance; 10. Timing. |  |  |
